# Supplementary material for: LDH-Co-Fe-Acetate: A New Efficient Sorbent for Azoic Dye Removal and Elaboration by Hydrolysis in Polyol, Characterization, Adsorption, and Anionic Exchange of Direct Red 2 as a Model Anionic Dye
Source: Materials (Basel). 2020 Jul 16;13(14):3183. doi: 10.3390/ma13143183 (PMC7411819; doi:10.3390/ma13143183)
Supplement: Supplementary file 1 [file materials-13-03183-s001.pdf]

materials

Supplementary Materials

LDH-Co-Fe-Acetate: A New Efficient Sorbent for Azoic Dye Removal and Elaboration by Hydrolysis in Polyol, Characterization, Adsorption, and Anionic Exchange of Direct Red 2 as a Model Anionic Dye

Nawal Drici-Setti [1,2,*], Paolo Lelli [3] and Noureddine Jouini [1,3,*]

1. Laboratoire des sciences des procédés et des matériaux (LSPM), Centre National de Recherche Scientifique (CNRS), Université Sorbonne Paris Nord, LSPM-CNRS-UPR 3407, 99 Avenue Jean-Baptiste Clément, 93430 Villetaneuse, France
2. Laboratoire de physico-chimie des matériaux, Département de Génie des Matériaux, Faculté de Chimie, université des Sciences et de Technologie-Mohamed Boudiaf d'Oran (USTO-MB), M'Nouar 1505, Oran 31000, Algeria
3. Département Hygiène, Sécurité, Environnement, Institut Universitaire de Technologie, Université Sorbonne Paris Nord, 8 Place du 8 mai 1945, 93200 Saint-Denis, France; labochimie@yahoo.fr (P.L.)

* Correspondance: drici_nawel@yahoo.fr (N.D.-S.); jouini@univ-paris13.fr (N.J.); Tel.: +213-41627176 (N.D.-S.); +331-49403435 (N.J.)

Received: 31 May 2020; Accepted: 14 July 2020; Published: 16 July 2020

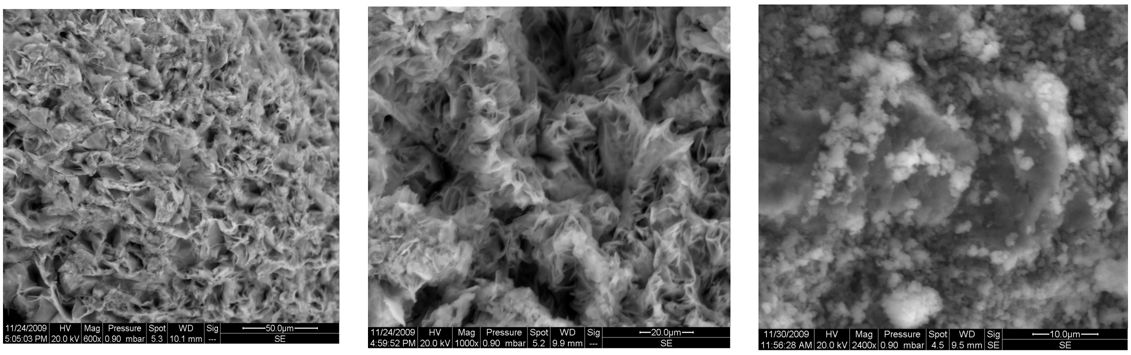

(**a**) (**b**) (**c**)

Figure S1. SEM images of (**a**) CoFe-Ac/$_{P}$, (**b**) CoFe-Ac/$_{Ex}$, (**c**) CoFe-CO_3/$_{A}$ LDHs.

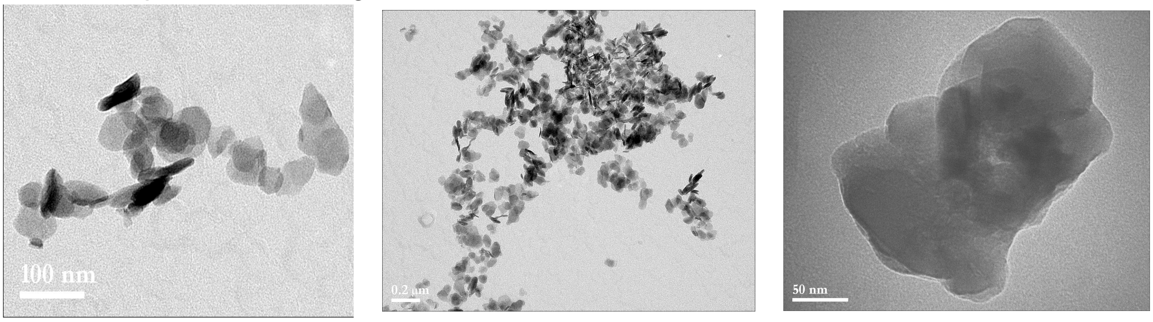

(**a**) (**b**) (**c**)

Figure S2. TEM images of **(a)** CoFe-Ac/$_{P}$, (**b**) CoFe-Ac/$_{Ex}$, **(c)** CoFe-CO_3/$_{A}$ LDHs.

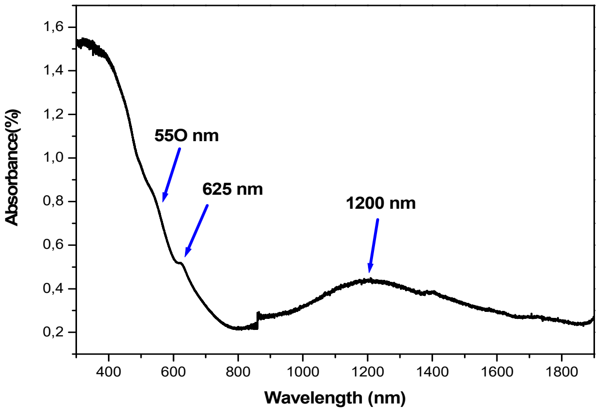

Figure S3. UV-Visible of of CoFe-Ac/$_{P}$ LDH.

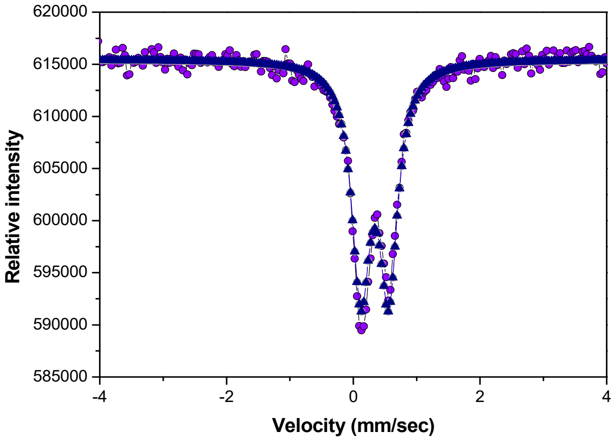

Figure S4. Mössbauer spectra of CoFe-Ac/$_{P}$ LDH.

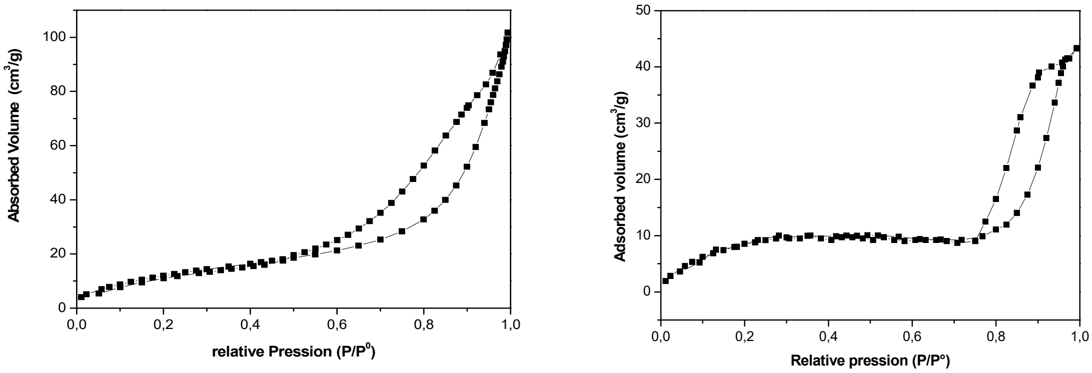

(a) **(b)**

Figure S5. N_2 adsorption–desorption isotherms of (**a**) CoFe-Ac/$_{P}$ (**b**) CoFe-Ac/$_{Ex}$ LDHs

Table S1. Textural properties of CoFe-Ac/$_{P}$ and CoFe-CO_3/$_{A}$ LDHs.

Compound	S_{BET} (m^2/g)	C_{BET}	Monolayer Volume V_m (cm^3/g)
CoFe-Ac/$_{P}$	48	22.78	11.17
CoFe-Ac/$_{Ex}$	50	24.02	11.52

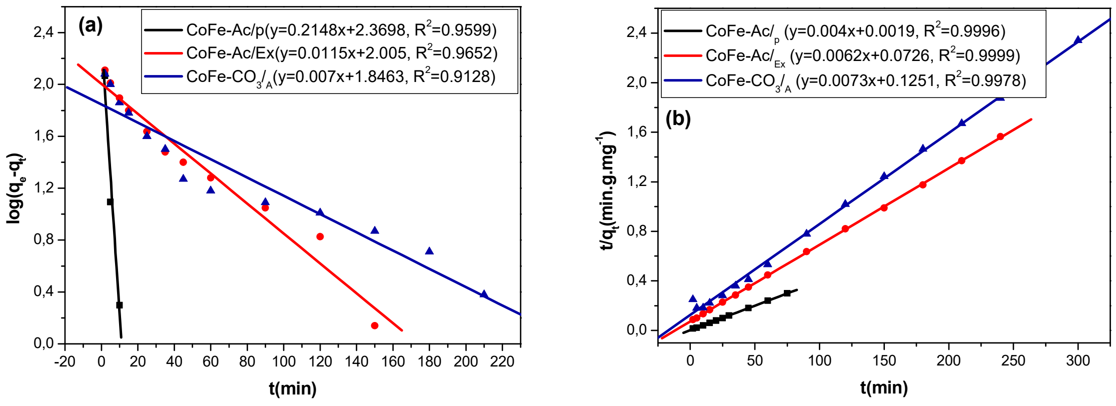

Figure S6. (**a**) Pseudo-first-order and (**b**) pseudo-second-order kinetics for adsorption of direct red 2 on CoFeAc/P, CoFe-Ac/Ex, and CoFe-CO_3/A LDHs.

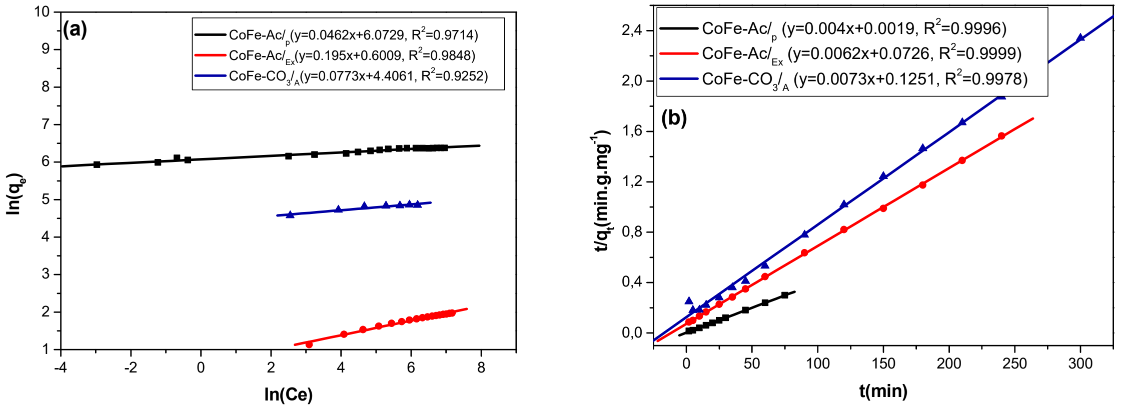

Figure S7. (**a**) Freundlich and (**b**) Langmuir isotherms for adsorption of direct red 2 on CoFe-Ac/P, CoFe-Ac/Ex, and CoFe-CO_3/A LDHs.

Toth model

Toth model was established from the Langmuir isotherm but by considering that the adsorption energy is not distributed homogeneously. Thus this model is of particular interest since it considers that the surface of the adsorbent is heterogeneous. In liquid phase, it is generally used like an adaptation of Langmuir model [1]. The Toth linear equation is given as:

$$\left(\frac{C_e}{q_e}\right)^{n} = \left(\frac{1}{q_m \times K_T}\right)^{n} + \left(\frac{1}{q_m}\right)^{n} \times (Ce)^{n} \quad (S1)$$

Where,

K_T is equilibrium Toth constant, Ce is the equilibrium concentration (mg/l), q_m is the maximum capacity of adsorption (mg/g) and n is the Toth exponent.

The constant of Toth K_T and the capacity of adsorption q_m are calculated from slop and intercept of the plot $(Ce/qe)^n = f(Ce)^n$.

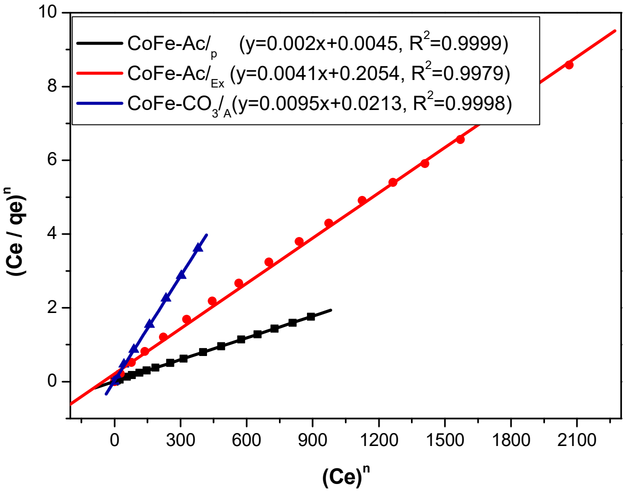

Figure S8. Toth isotherm for adsorption of direct red 2 on CoFe-Ac/P, CoFe-Ac/Ex, and CoFe-CO_3/A LDHs.

Table S2. Toth isotherm parameters for the adsorption of direct red 2 onto LDH samples.

Toth Isotherm	q_m (mg/g)	K_L	R^2
CoFe-Ac/p	578.77	0.436	0.9999
CoFe-Ac/Ex	175.49	0.0252	0.9979
CoFe-CO_3/A	128.125	0.431	0.9998

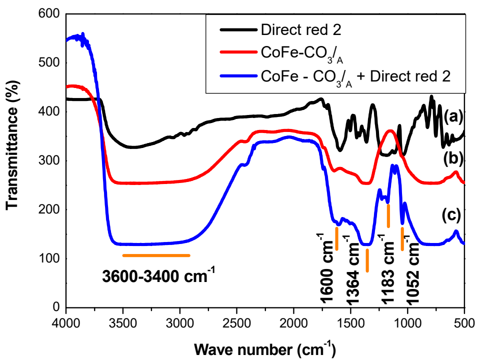

Figure S9. FT-IR spectra before and after adsorption for CoFe-CO_3/A.

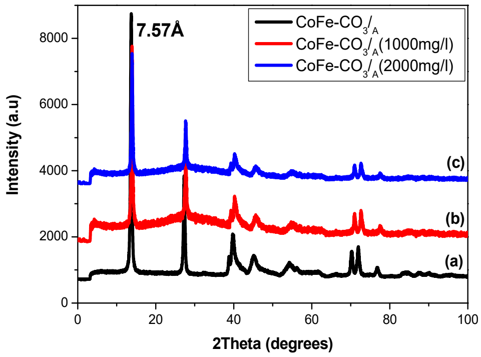

Figure S10. X-ray diffractogram before and after adsorption for $CoFe-CO_3/_A$.

Reference

1. *Toth, J*. Gas-(Dampf-)Adsorption auf festen Oberflecken inhomogener Aktivität, *III*, *Acta Chimica Academiae Scientarium Hungaricae*, **1962**, *32*, *39*.

© 2020 by the authors. Licensee MDPI, Basel, Switzerland. This article is an open access article distributed under the terms and conditions of the Creative Commons Attribution (CC BY) license (http://creativecommons.org/licenses/by/4.0/).
